# Supplementary material for: Chromosome-Level Genome Assembly of a Human Fungal Pathogen Reveals Synteny among Geographically Distinct Species
Source: mBio. 2022 Jan 4;13(1):e02574-21. doi: 10.1128/mbio.02574-21 (PMC8725592; doi:10.1128/mbio.02574-21)
Supplement: TABLE S1 [file mbio.02574-21-st001.docx]

**Table S1A.** **Statistics of previous and current *Histoplasma* genome assemblies.** Completeness of the genome assemblies was assessed using BUSCO version 4.0.4, with dataset eurotiomycetes_odb10 [(53)](https://paperpile.com/c/3s2Y8c/tSCo). Statistics and percentage of complete BUSCO groups are shown for previously published genomes and the assemblies reported in this study.

| **Strain** | **Source** | **Method** | **Length** | **Contigs** | **Ctg N50** | **Ctg L50** | **Scaffolds** | **Sc N50** | **Sc L50** | **BUSCO** |
| --- | --- | --- | --- | --- | --- | --- | --- | --- | --- | --- |
| WU24 | BROAD | Sanger | 33,030,326 | 2,873 | 27,346 | 233 | 280 | 2,885,091 | 4 | 87.4% |
| WU24 | This study | Oxford Nanopore | 32,531,515 | 8 | 5,328,076 | 3 | -- | -- | -- | 98.8% |
| G217B | WUSTL | Sanger | 39,270,953 | 261 | 634,562 | 21 | -- | -- | -- | 98.6% |
| G217B | This study | Oxford Nanopore | 39,447,273 | 12 | 6,680,169 | 3 | -- | -- | -- | 98.6% |
| G186AR | BROAD | Sanger | 30,483,324 | 359 | 191,715 | 48 | 88 | 1,769,553 | 6 | 97.4% |
| G186AR | This study | Oxford Nanopore | 31,111,494 | 7 | 5,585,295 | 3 | -- | -- | -- | 98.8% |
| G184AR | This study | Oxford Nanopore | 30,991,520 | 12 | 4,063,218 | 3 | -- | -- | -- | 98.8% |
| H88 | BROAD | Sanger | 37,943,219 | 464 | 168,190 | 67 | 17 | 5,001,999 | 4 | 97.7% |
| H88 | This study | Oxford Nanopore | 37,996,987 | 7 | 7,000,705 | 3 | -- | -- | -- | 98.9% |
| H143 | BROAD | Sanger | 38,961,716 | 4,267 | 15,865 | 663 | 49 | 1,740,271 | 7 | 85.0% |

**Table S1B. Statistics of annotation transfer for the new *Histoplasma* genome assemblies.** Number of transcripts that either failed to map by BLAT to the new assembly (Not mapped), mapped but with a change in coding sequence length (CDS len), with a change in coding sequence (CDS seq), with a change in UTR length (Exon length), a change in UTR sequence (Exon seq), or with no changes to CDS or UTR (Match). Each transcript is recorded in the leftmost applicable column, such that the sum over each row gives the total previously annotated transcripts for that genome.

| **Genome** | **Not mapped** | **CDS len** | **CDS seq** | **Exon length** | **Exon seq** | **Match** |
| --- | --- | --- | --- | --- | --- | --- |
| WU24 | 54 (1%) | 385 (4%) | 1397 (15%) | 187 (2%) | 145 (2%) | 7080 (77%) |
| G217B | 31 (0%) | 12 (0%) | 443 (4%) | 843 (7%) | 104 (1%) | 10880 (88%) |
| G186AR | 11 (0%) | 5 (0%) | 348 (3%) | 529 (4%) | 123 (1%) | 11647 (92%) |
| G184AR | 31 (0%) | 10 (0%) | 527 (4%) | 819 (6%) | 117 (1%) | 11159 (88%) |
| H88 | 15 (0%) | 15 (0%) | 457 (4%) | 546 (4%) | 80 (1%) | 11062 (91%) |
