# Supplementary figures and images for: Chromosome-Level Genome Assembly of a Human Fungal Pathogen Reveals Synteny among Geographically Distinct Species
Source: mBio. 2022 Jan 4;13(1):e02574-21. doi: 10.1128/mbio.02574-21 (PMC8725592; doi:10.1128/mbio.02574-21)

Figure S1

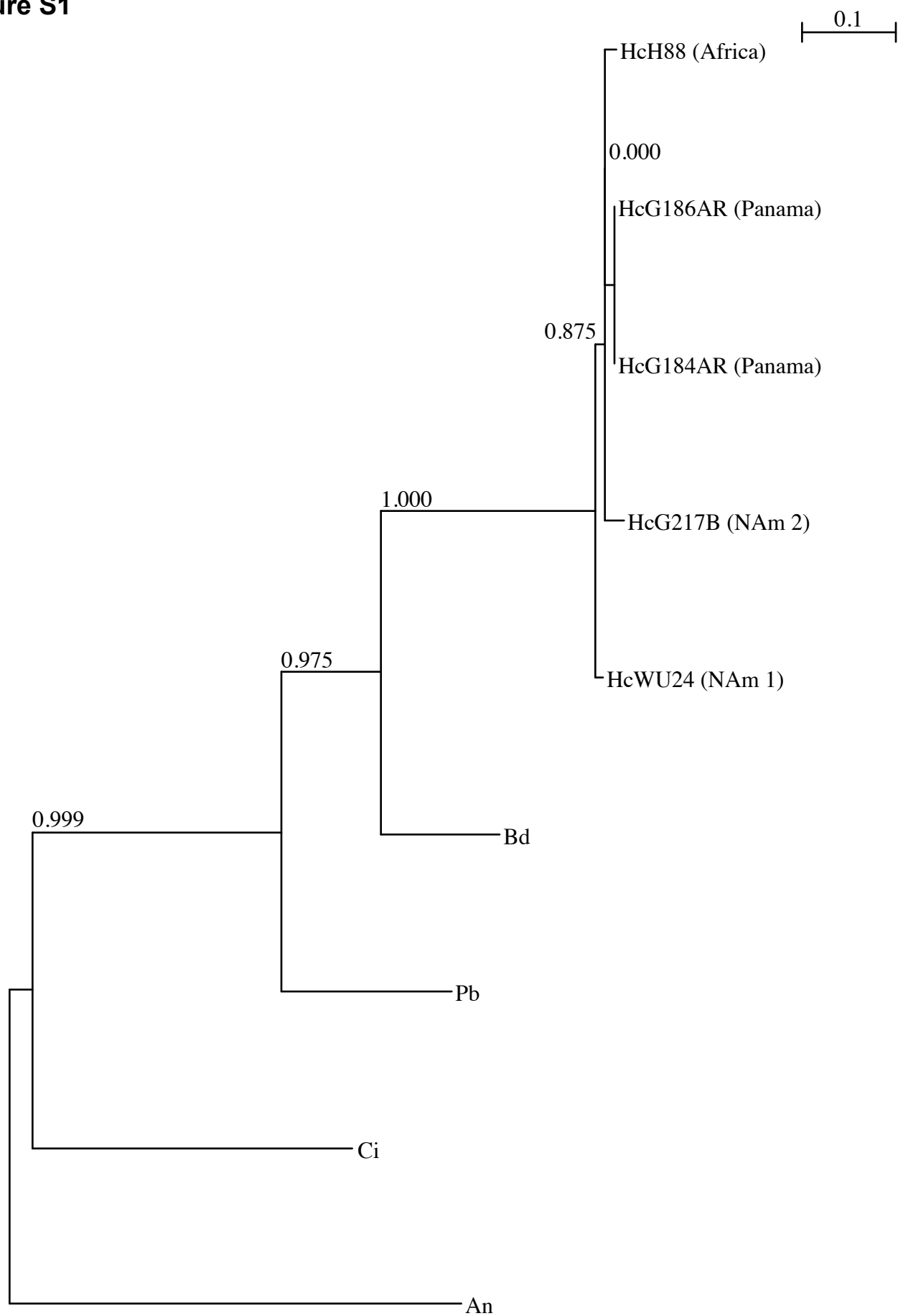

Supplement: FIG S1 [file mbio.02574-21-sf001.pdf]

Figure S4A

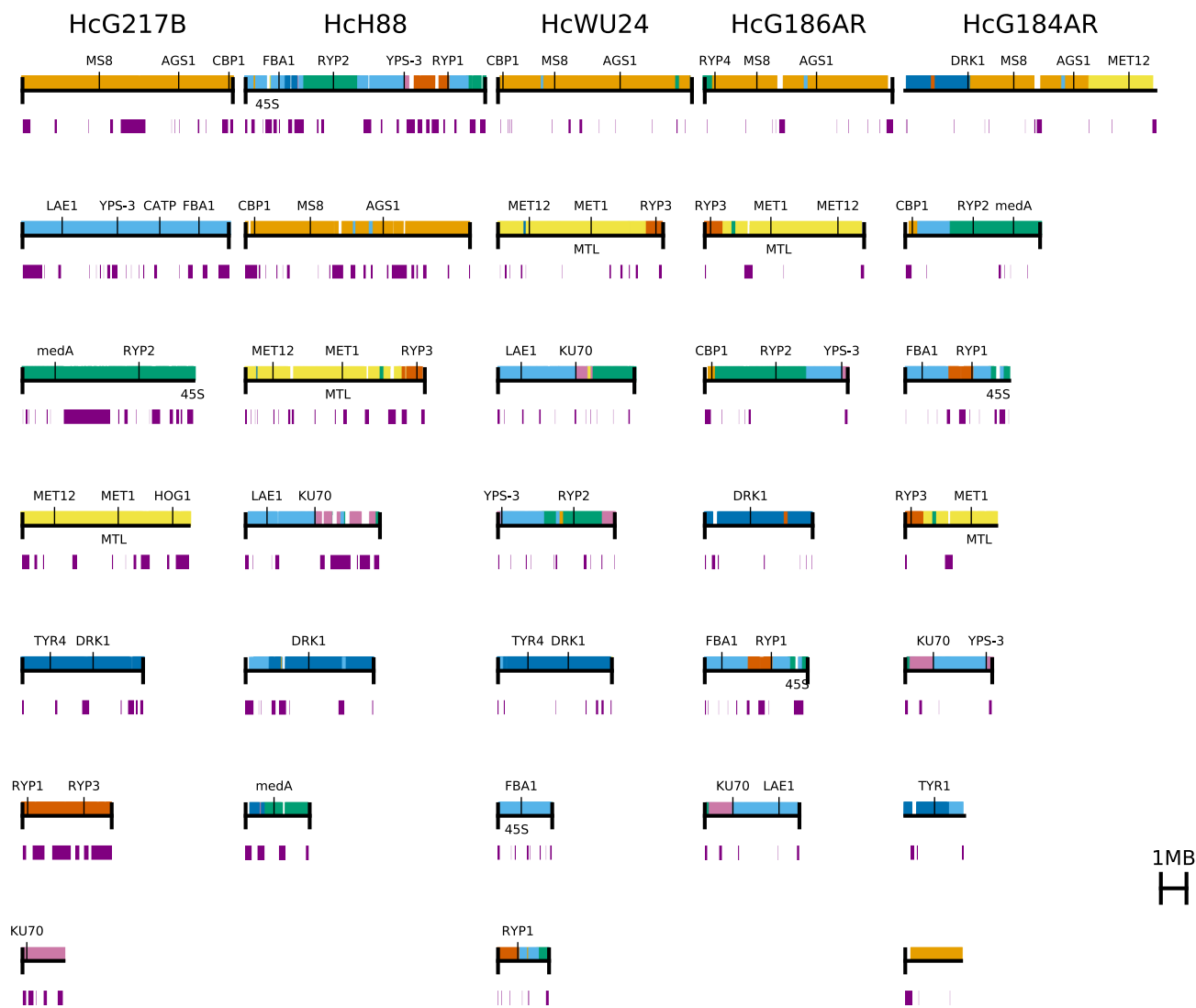

Figure S4B

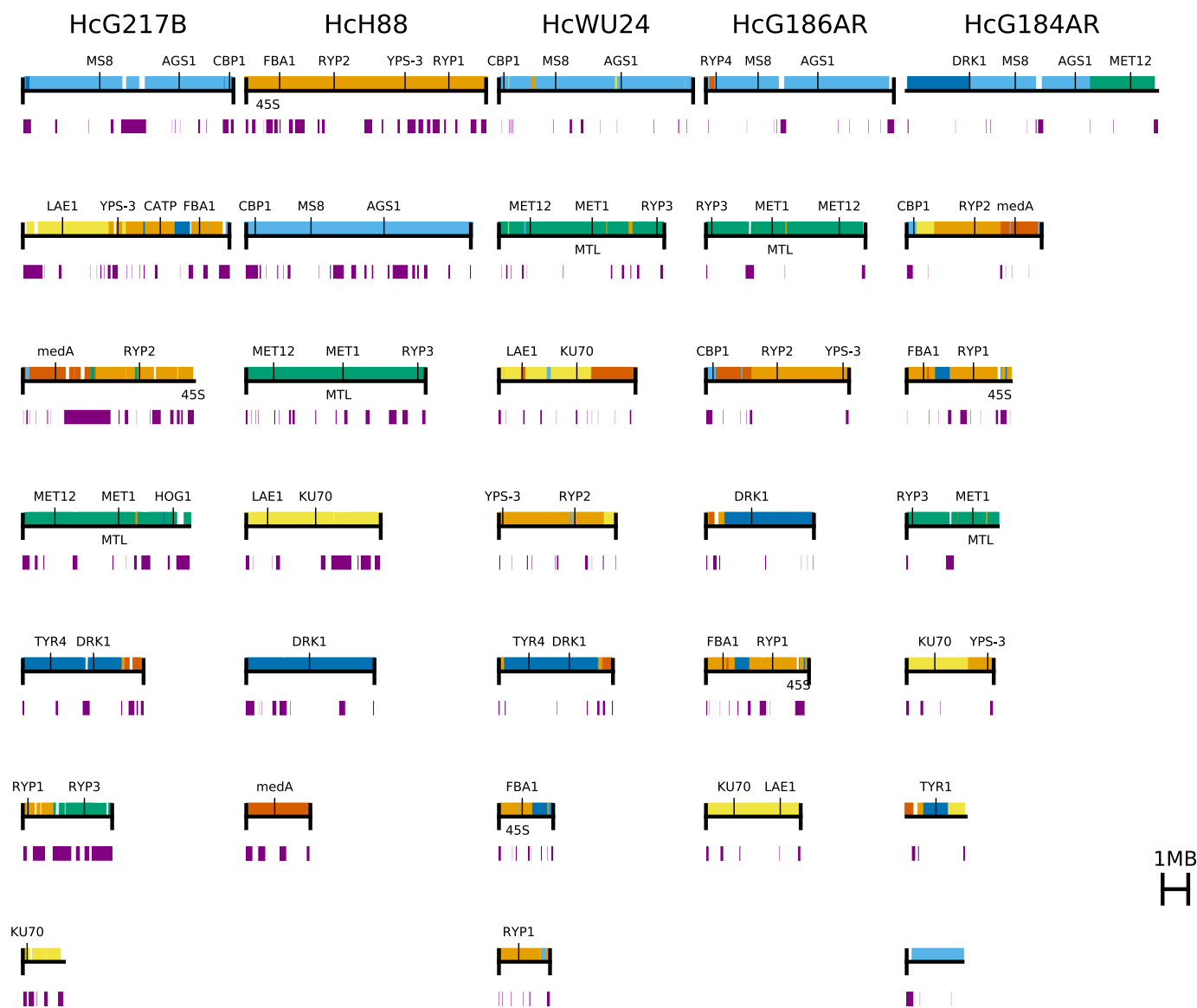

Figure S4C

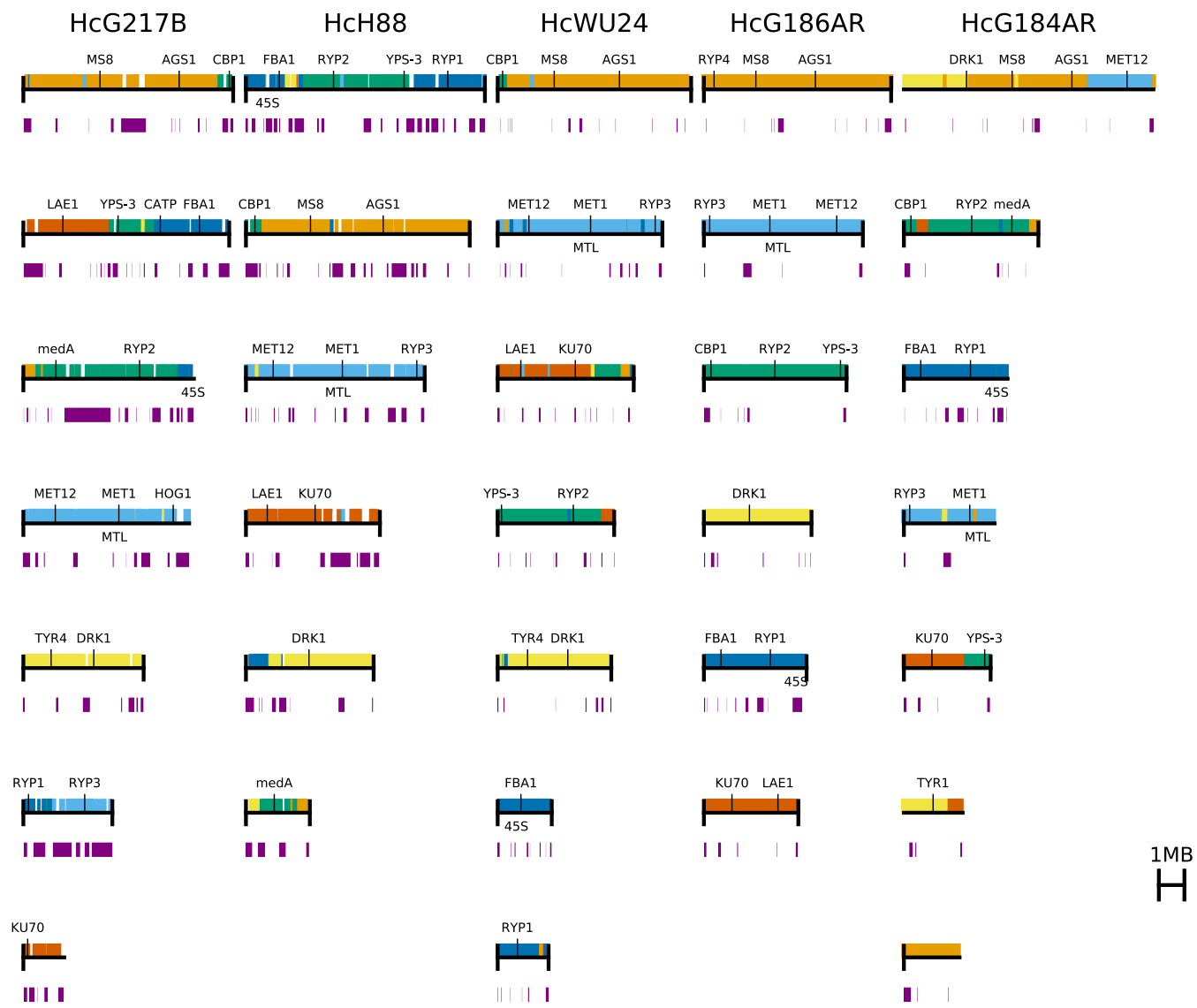

Figure S4D

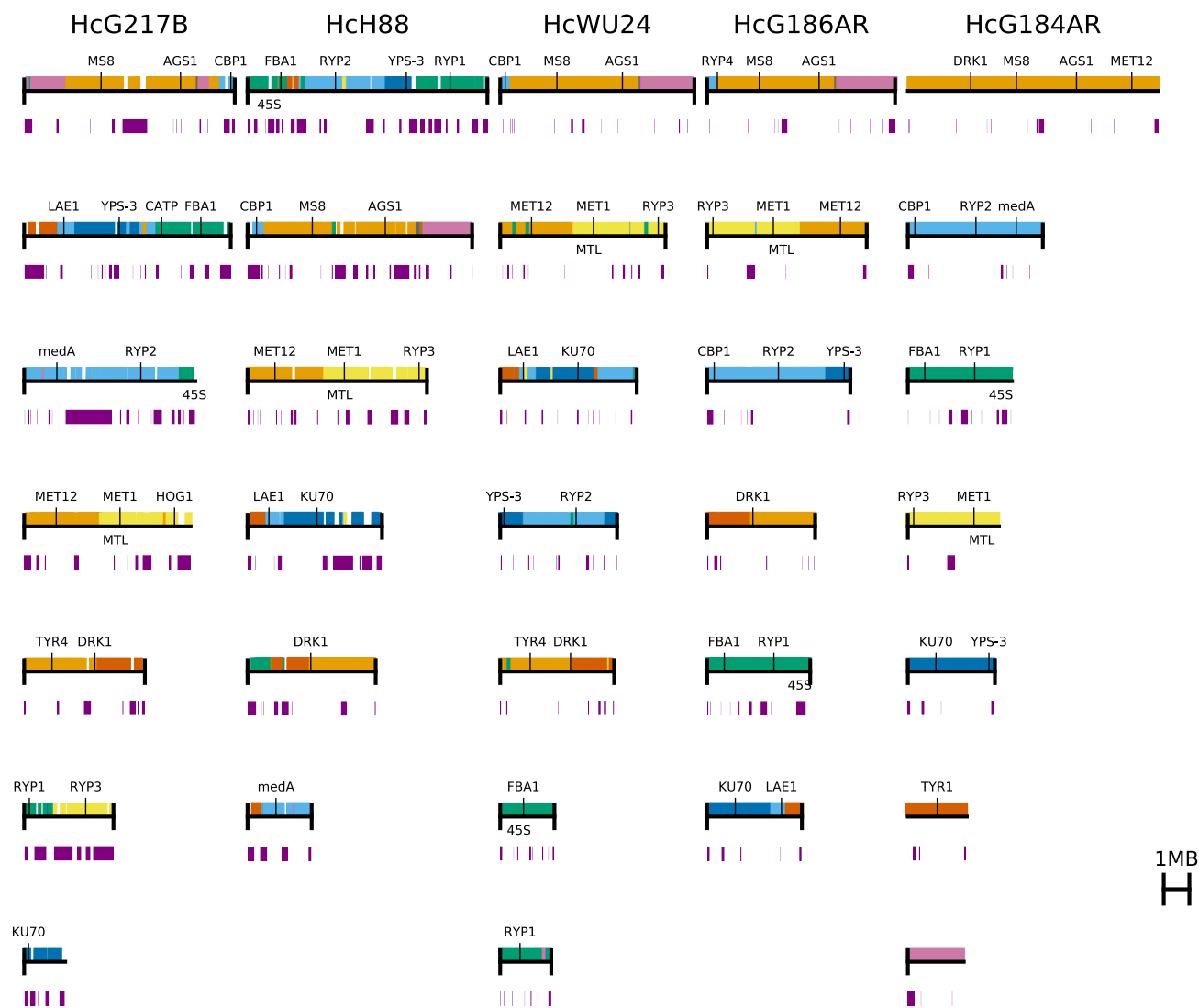

Supplement: FIG S4 [file mbio.02574-21-sf004.pdf]

WU24

WU24

G217B

H88

G186AR

G184AR

SH01

SHO1

SH01

SHO1

SHO1

100KB

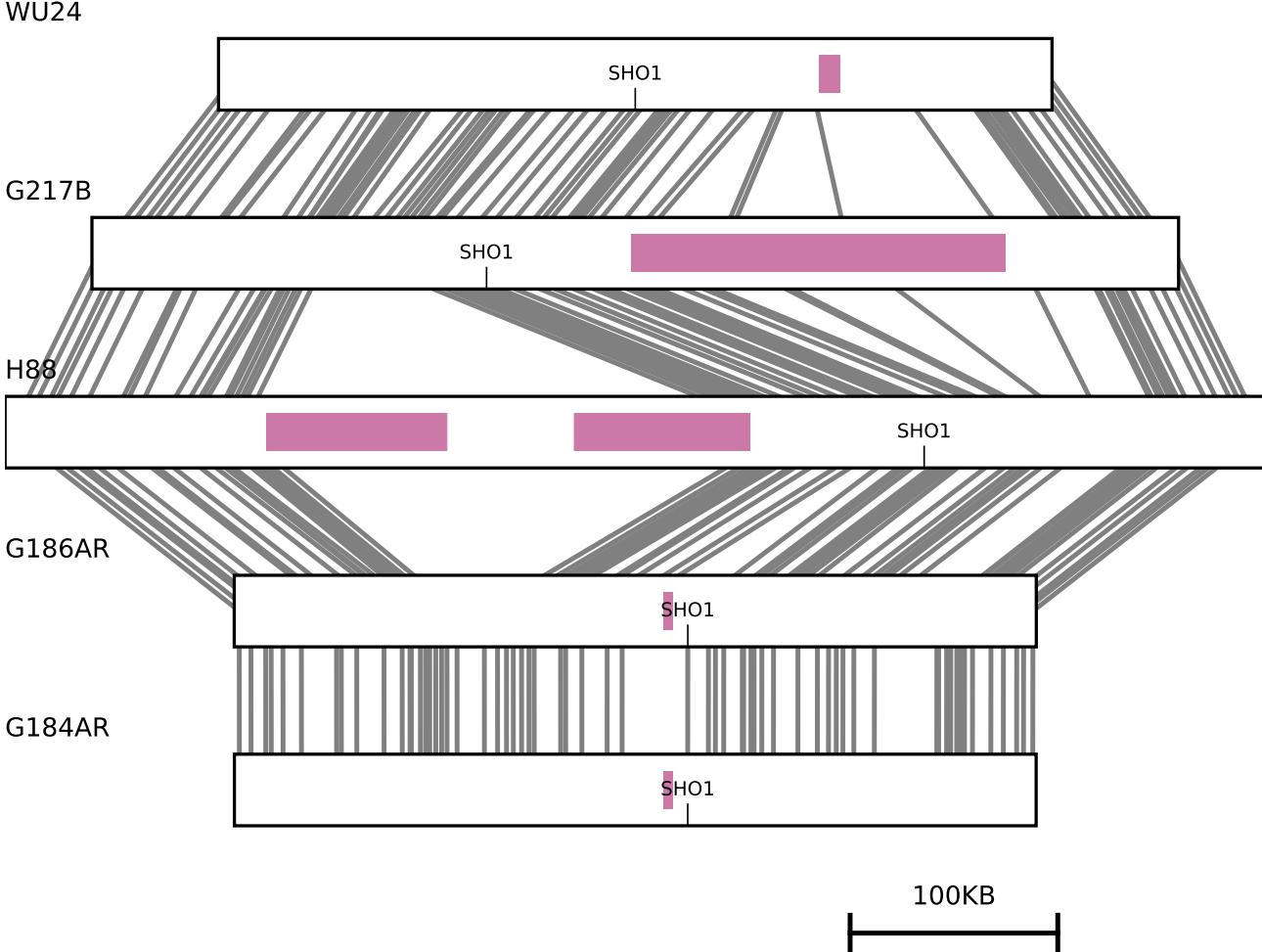

Figure S5B

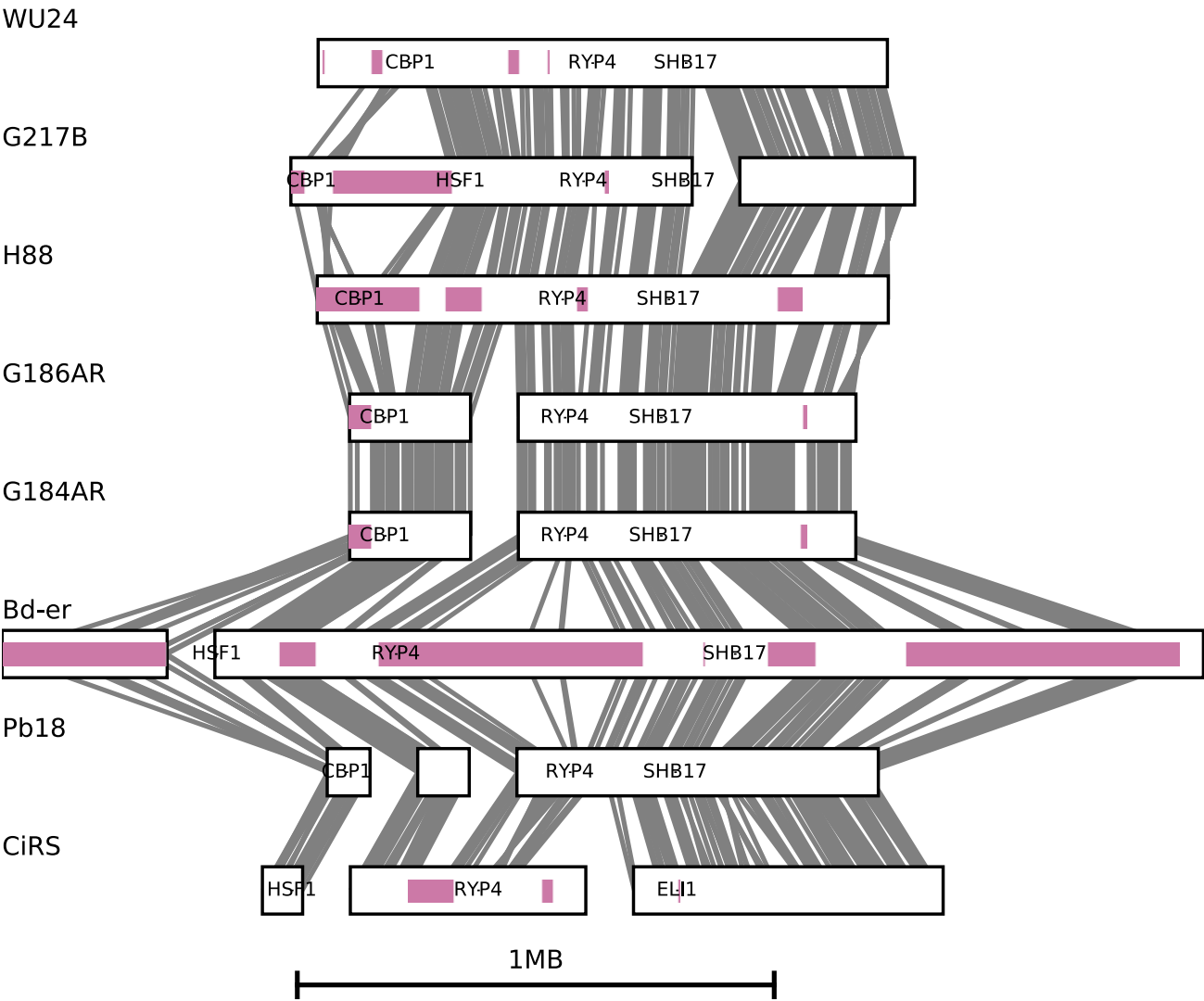

Figure S5C

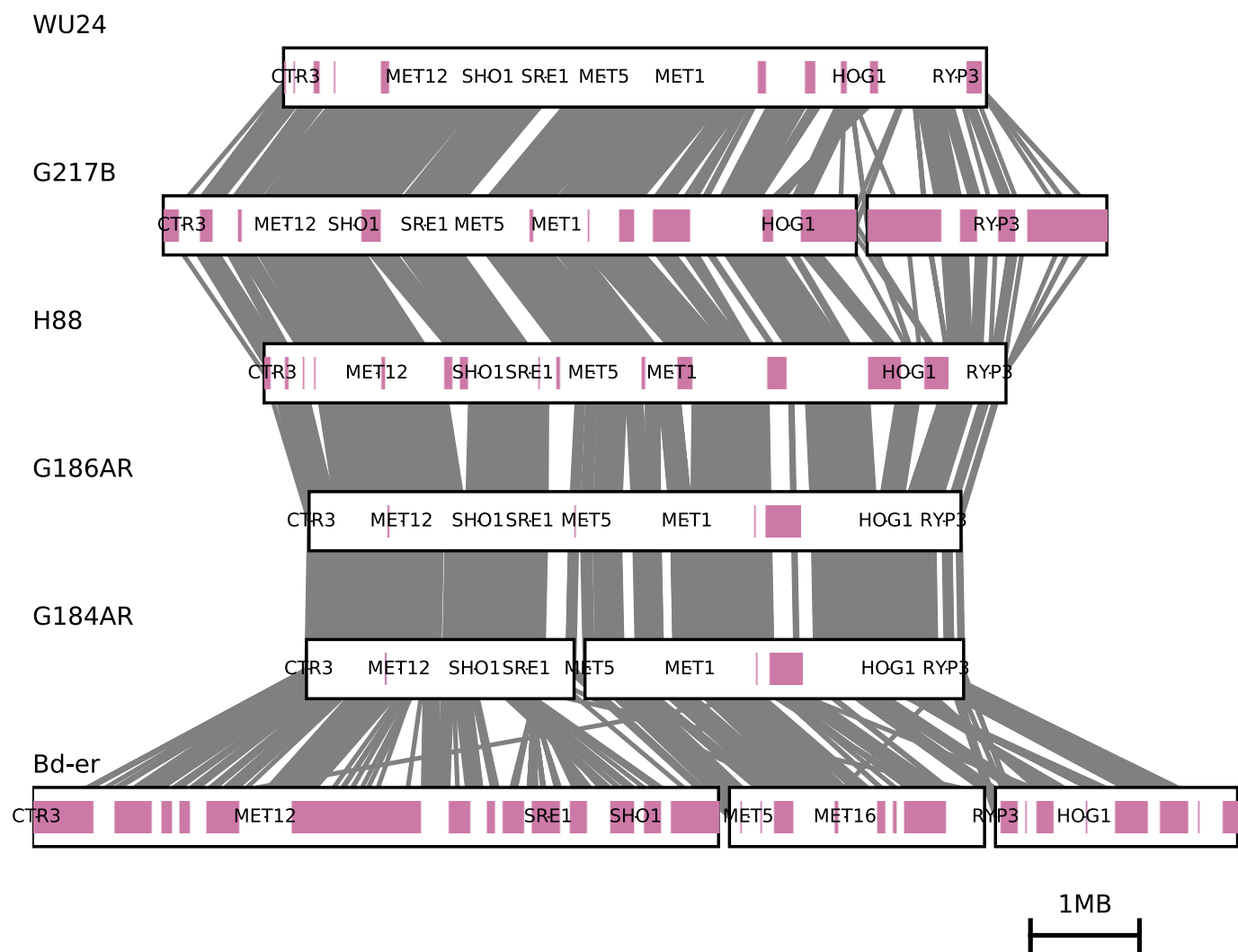

Figure S5D

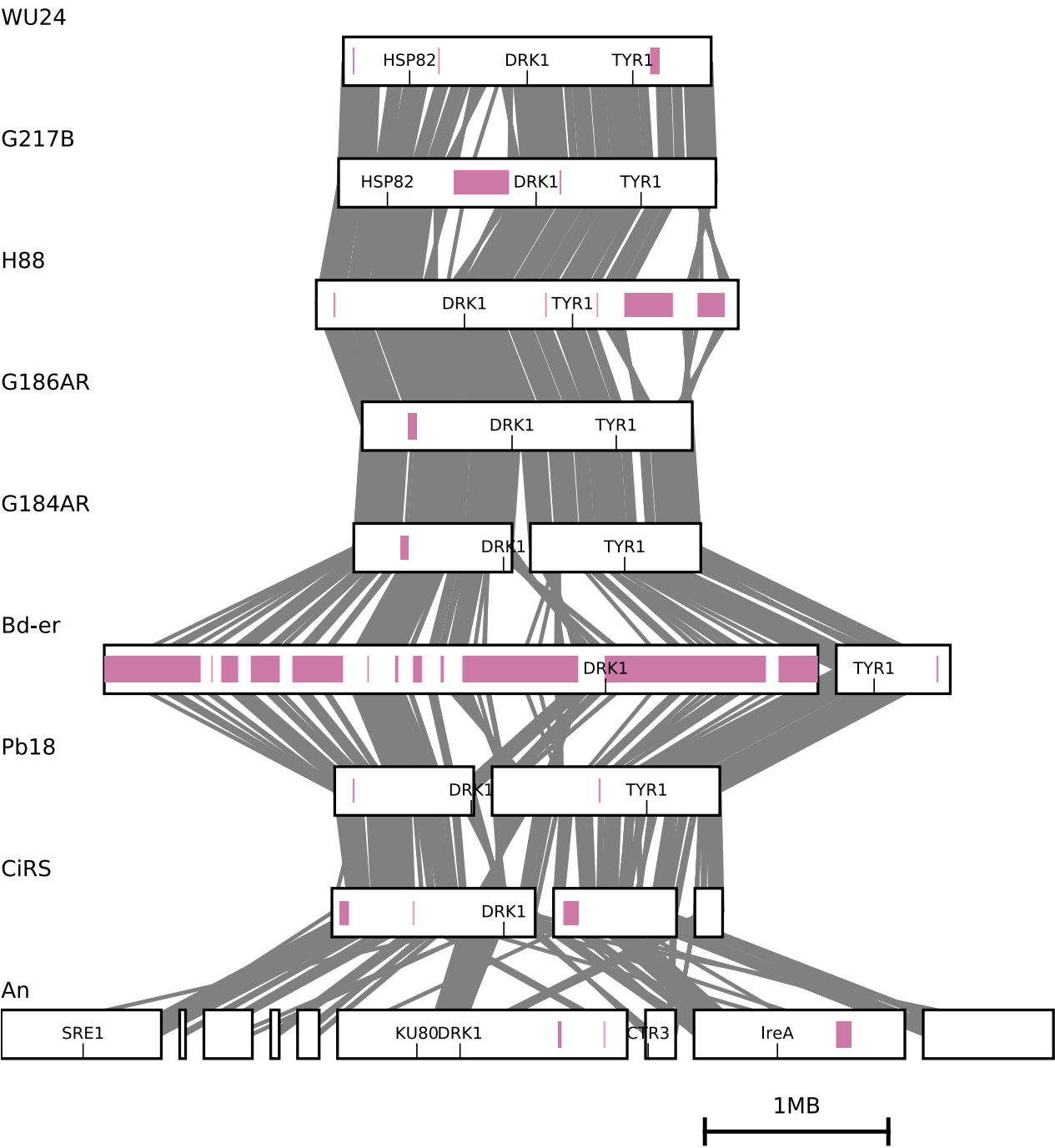

Supplement: FIG S5 [file mbio.02574-21-sf005.pdf]

Figure S6A

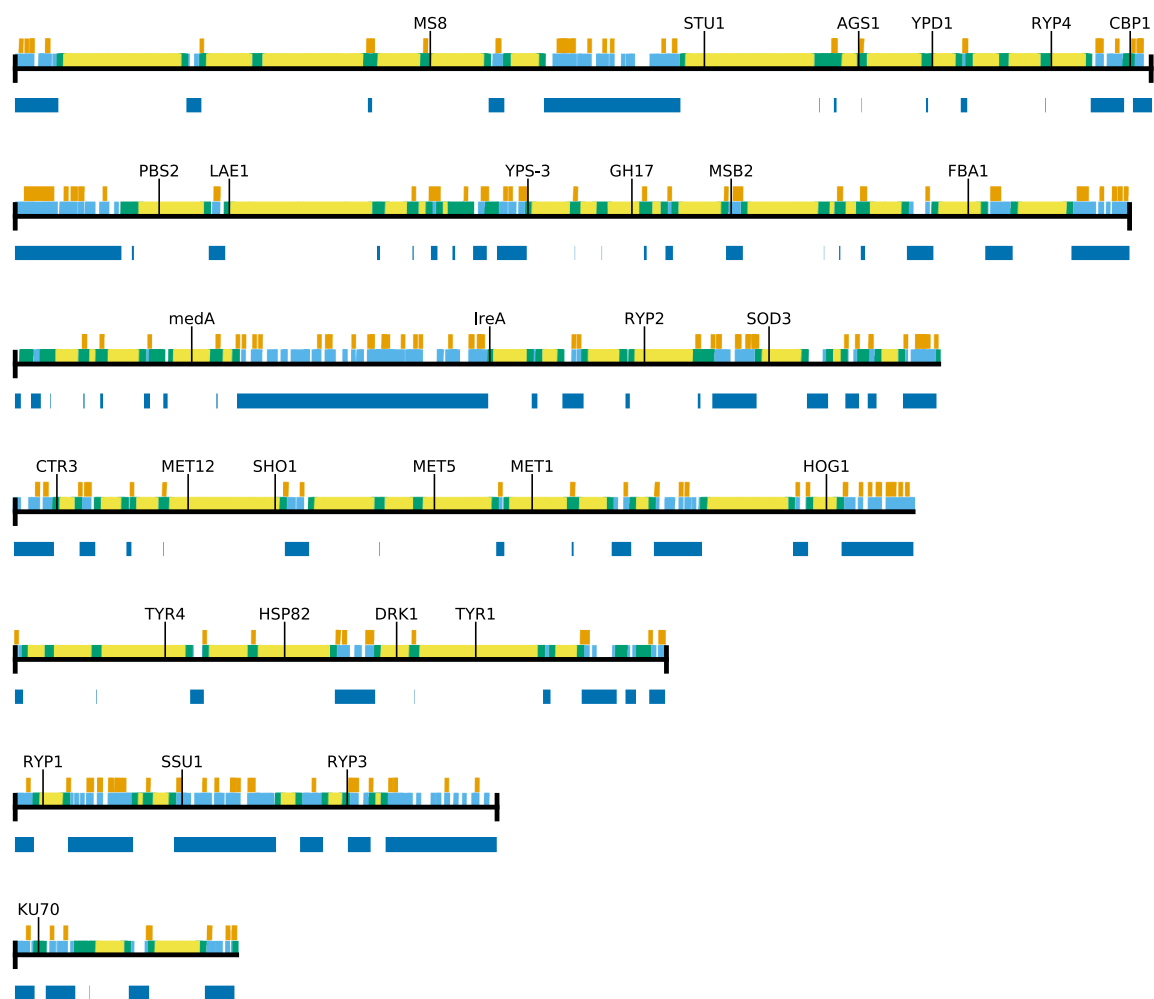

Figure S6B

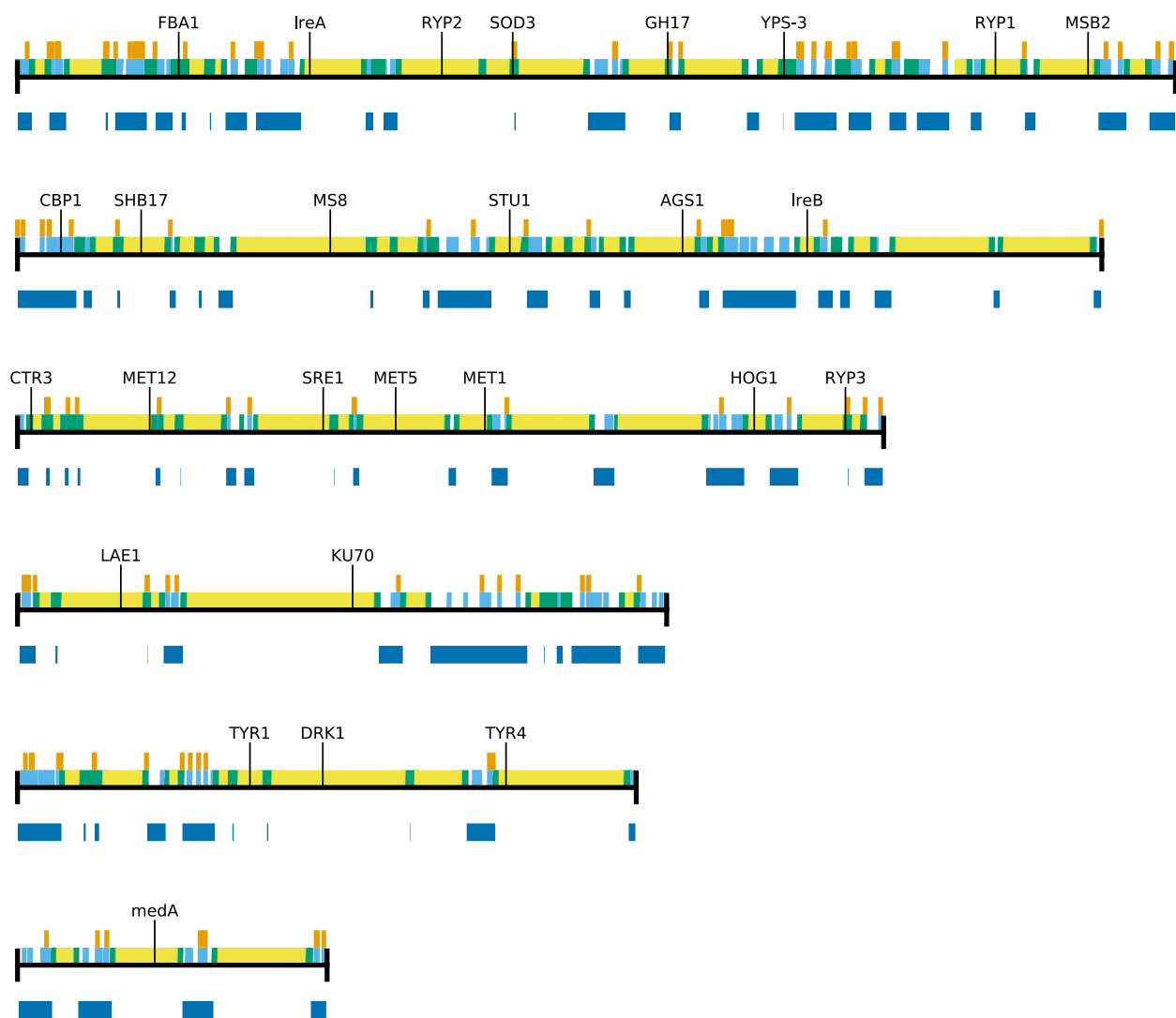

Figure S6C

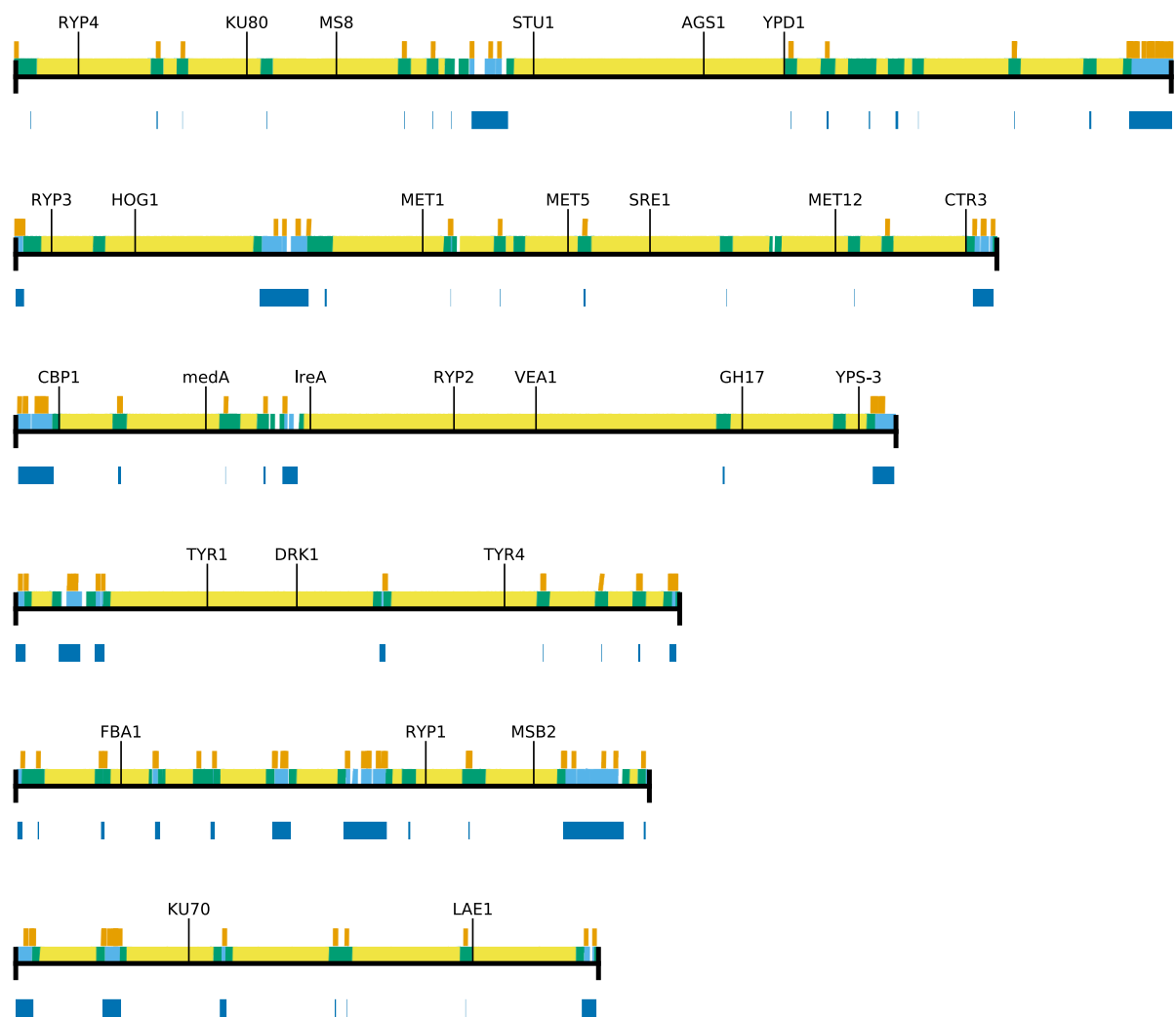

Supplement: FIG S6 [file mbio.02574-21-sf006.pdf]

Figure S7

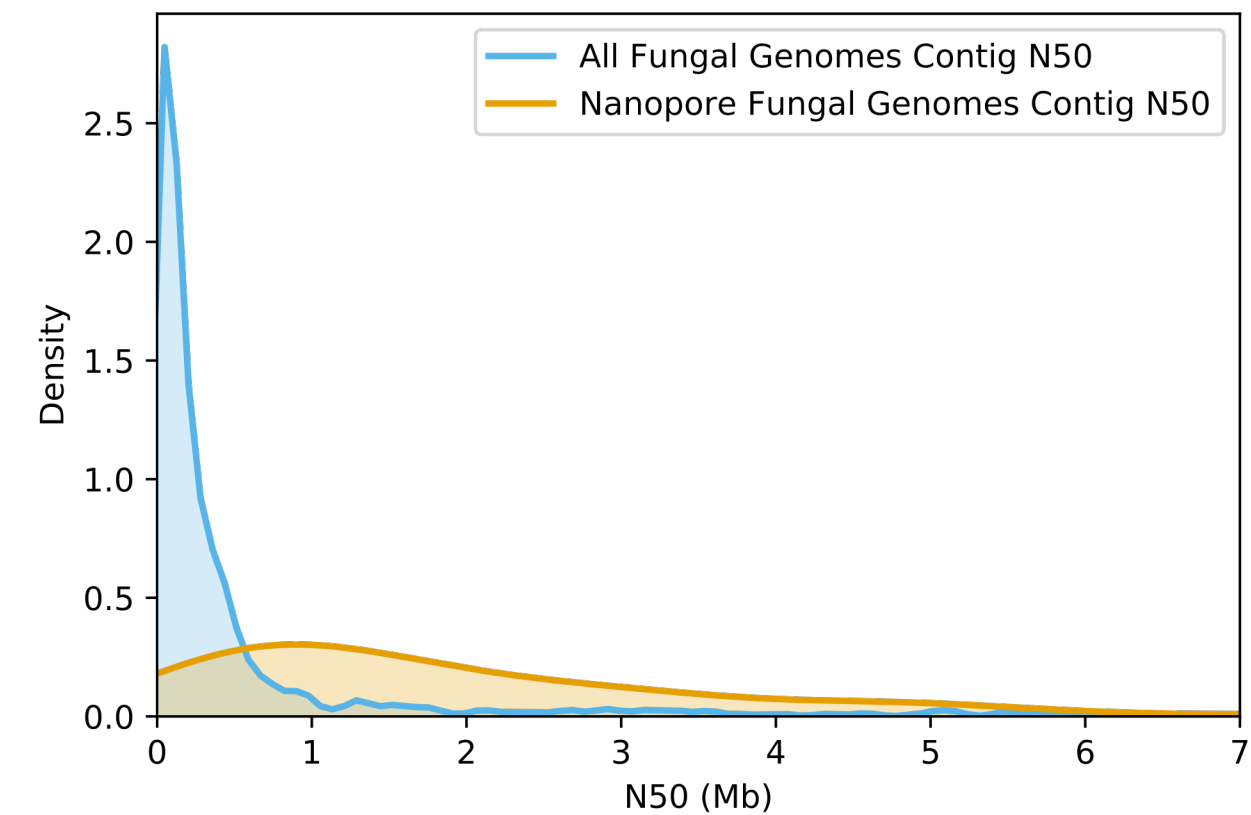

Supplement: FIG S7 [file mbio.02574-21-sf007.pdf]

Figure S2

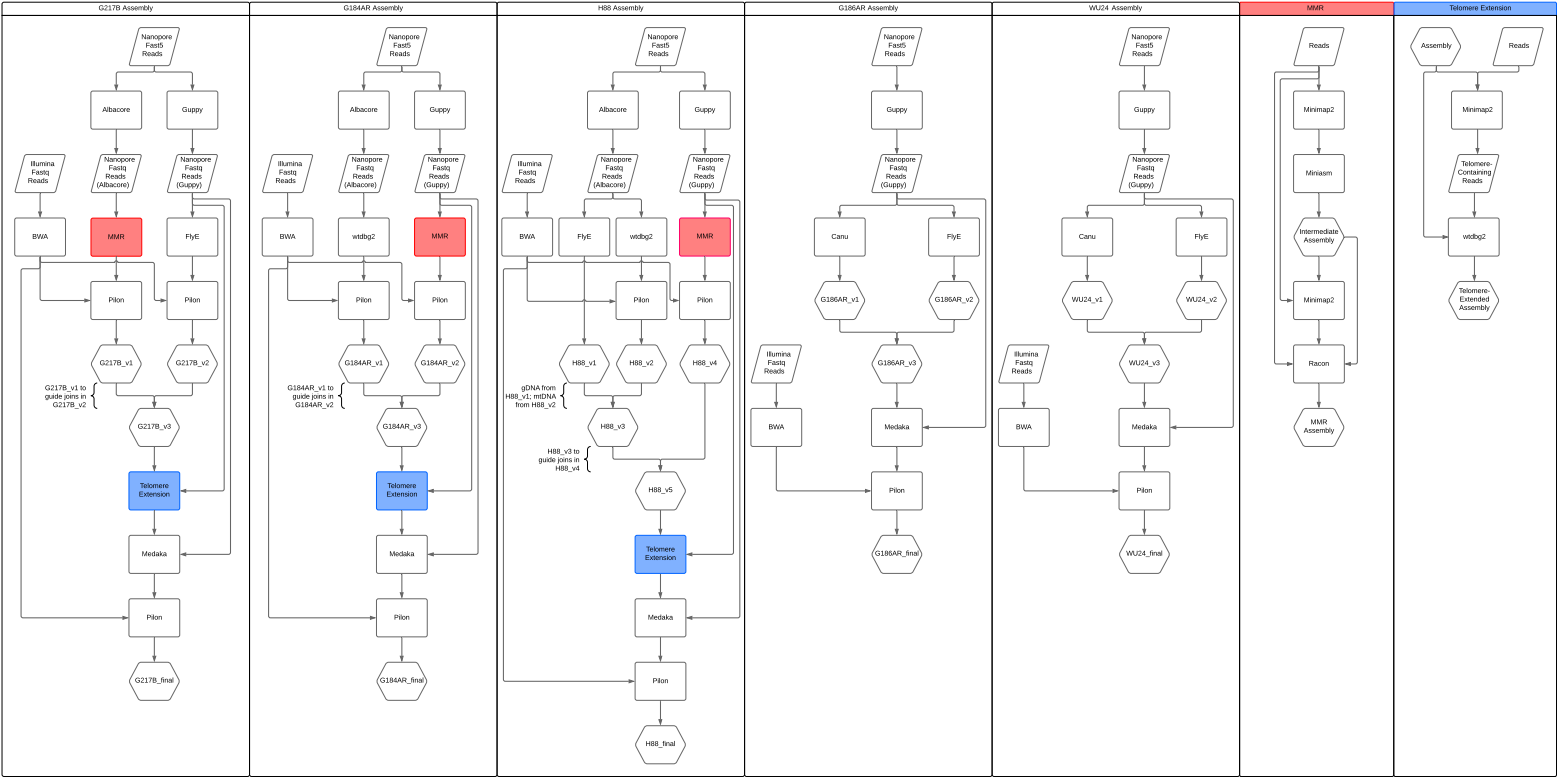

Supplement: FIG S2 [file mbio.02574-21-sf002.pdf]

Figure S3

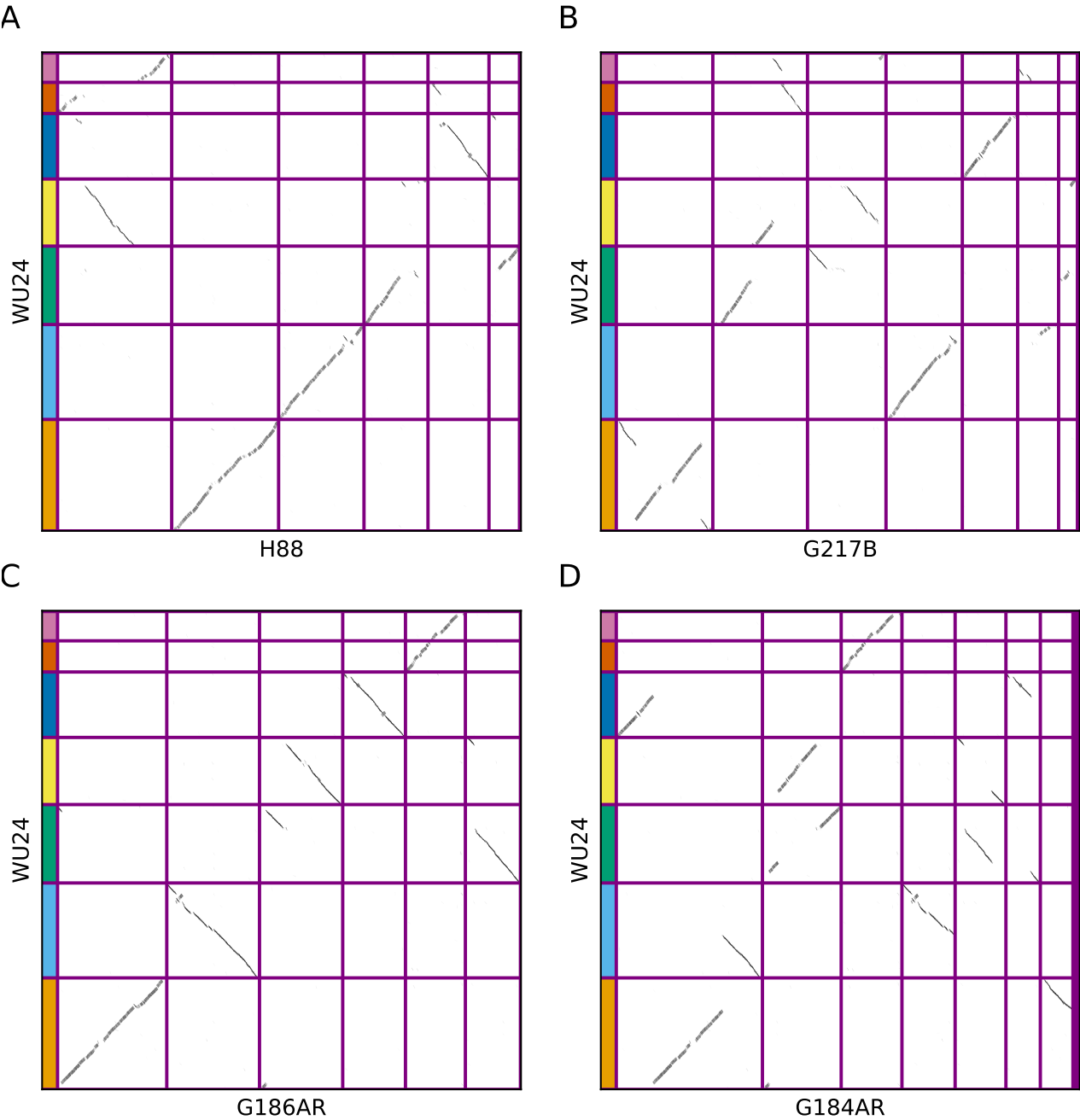

Supplement: FIG S3 [file mbio.02574-21-sf003.pdf]
