# Supplementary material for: Chromosome-Level Genome Assembly of a Human Fungal Pathogen Reveals Synteny among Geographically Distinct Species
Source: mBio. 2022 Jan 4;13(1):e02574-21. doi: 10.1128/mbio.02574-21 (PMC8725592; doi:10.1128/mbio.02574-21)
Supplement: TABLE S2 [file mbio.02574-21-st002.docx]

**Table S2. Analysis of repeat regions in *Histoplasma* genomes.** Transposon statistics for the ONT genome assemblies are shown. Total = total base pairs, Repeat = number of base pairs spanned by union of TBLASTN and LTRHarvest annotations, Annealed = number of base pairs spanned by LTR-rich blocks (*c.f.* Fig. 3A), T-R = Total - Repeat, T-A = Total - Annealed, R% = Repeat/Total, A% = Annealed/Total, RL = number of repeat annotations (after union), AL = number of LTR-rich blocks, tgenes = number of transposon genes, rgenes = number of transposon-embedded genes, agenes = number of transposon-adjacent genes, %r = rgenes/(total genes), %a = agenes/(total genes).

| **Genome** | **Total** | **Repeat** | **Annealed** | **T-R** | **T-A** | **R%** | **A%** | **RL** | **AL** | **tgenes** | **rgenes** | **agenes** | **%r** | **%a** |
| --- | --- | --- | --- | --- | --- | --- | --- | --- | --- | --- | --- | --- | --- | --- |
| HcG217B | 39447273 | 7911417 | 13807944 | 31535856 | 25639329 | 0.2 | 0.35 | 1991 | 95 | 12282 | 371 | 1133 | 0.03 | 0.09 |
| HcH88 | 37996987 | 5892053 | 11804992 | 32104934 | 26191995 | 0.16 | 0.31 | 1609 | 87 | 12160 | 165 | 775 | 0.01 | 0.06 |
| HcWU24 | 32531515 | 1148983 | 3213981 | 31382532 | 29317534 | 0.04 | 0.1 | 387 | 128 | 9194 | 45 | 403 | 0 | 0.04 |
| HcG186AR | 31111494 | 1717918 | 3400858 | 29393576 | 27710636 | 0.06 | 0.11 | 493 | 70 | 12652 | 202 | 484 | 0.02 | 0.04 |
| HcG184AR | 30991520 | 1710517 | 3311154 | 29281003 | 27680366 | 0.06 | 0.11 | 480 | 69 | 12632 | 202 | 449 | 0.02 | 0.04 |
